# Minimal Dosage of Porcine Circovirus Type 2d Based Virus-like Particles to Induce Stable Protective Immunity against Infection

**DOI:** 10.3390/pathogens10121644

**Published:** 2021-12-20

**Authors:** Jong-Hyuk Baek, Sang-Ho Cha, Sun-Hee Cho, Myung-Shin Lee, Changhoon Park

**Affiliations:** 1 Department of Animal Vaccine Development, BioPOA, Dongtangiheung-ro, Hwaseong-si 18469, Korea; kagala05@nate.com (J.-H.B.); sfzen@biopoa.co.kr (S.-H.C.); 2 PRRS Research Laboratory, Viral Diseases Division, Animal and Plant Quarantine Agency, Gimcheon 39660, Korea; virusmania@korea.kr; 3 Department of Microbiology and Immunology, Eulji University School of Medicine, Yongdu-dong, Junggu, Daejeon 34824, Korea; imslee@gmail.com

**Keywords:** porcine circovirus, virus like particle, vaccine, minimal dosage, immunity

## Abstract

In recent years, porcine circovirus type 2d (PCV2d) has achieved a dominant position worldwide. Various PCV2d capsid-based vaccines have been used to alleviate concerns regarding the emergence of the variant. This study aimed to determine the dosage of recombinant PCV2d capsid protein to induce protective efficacy against experimental challenge with a virulent PCV2d strain. Conventional 3-week-old pigs were intramuscularly inoculated with different doses of the protein (60, 20, 10 and 2 µg). Four weeks after vaccination, all pigs were challenged with pathogenic PCV2d (SNU140003), which was isolated from a farm severely experiencing PCV2-associated disease in Korea. Vaccination with greater than 10 µg of the capsid protein caused a significant (*p <* 0.05) reduction in PCV2d viremia, lymphoid lesions and lymphoid PCV2 antigen levels in vaccinated challenged pigs compared to unvaccinated challenged pigs. The vaccination also resulted in significantly higher (*p <* 0.05) titers of neutralizing antibodies against PCV2d. However, the pigs vaccinated with 2 µg had significantly lower neutralizing antibody titers than the other vaccinated groups. They showed a similar level of challenged PCV2d in serum and lymphoid lesion score compared to unvaccinated challenged pigs. The difference in efficacy among the vaccinated groups indicates that there may be a baseline dosage to induce sufficient neutralizing antibodies to prevent viral replication in pigs. In conclusion, at least 10 µg dosage of capsid protein is essential for stable protective efficacy against PCV2d in a pig model.

## 1. Introduction

Porcine circovirus type 2 virus (PCV2)-associated disease (PCVAD) is one of the most severe threats to the swine industry. In growing pigs, the symptoms can be summarized with a variety of clinical manifestations, including excessive weight loss, respiratory illness and increased mortality [1,2]. Post-weaning multisystemic wasting syndrome and porcine dermatitis and nephropathy syndrome have also been associated with PCVAD [3,4,5]. Based on numerous etiological and pathological evidence, PCV2 has been an obligatory agent for PCVAD, but several other infectious and noninfectious factors have also been reported to contribute to the manifestation of the symptoms through various mechanisms [6].

PCV2 has a high mutation rate comparable to that of RNA viruses [7,8], which may further promote the rapid evolution and emergence of unique PCV2 genotypes. Since the initial commercial vaccine was released, the evolutionary history of this virus has clearly reflected this fact. PCV2a was the predominant genotype among the PCV2 variants when the initial vaccine was being developed [9,10]. However, after the first commercial PCV2a-based vaccine was launched in 2007, PCV2b supplanted PCV2a as the predominant genotype worldwide to avoid vaccine immunity [11,12,13]. Recently, another genotypic shift has occurred with PCV2d becoming the predominant strain in the global pig population, replacing PCV2a and PCV2b [14]. In terms of an outbreak of PCVAD caused by PCV2d in PCV2a-vaccinated herds, this second shift has raised questions about the efficacy of the PCV2a vaccine against the PCV2d genotype [15,16].

Most PCV2 vaccines belong to two categories: subunit or inactivated virus vaccines [17]. The two vaccine types have virus-like particles (VLPs) in common as major antigens, which consist of the viral capsid. VLP is sufficiently effective to induce antibodies to neutralize several genotypes of PCV2. Current commercial VLP vaccines have been shown to protect post-weaning and growing pigs against experimental challenge with not only PCV2a but also other genotype strains (PCV2b and PCV2d) [15,18]. Despite continuous doubt, PCV2a VLPs seem to provide sufficient immunity to pigs under experimental conditions [19,20].

Generally, the efficacy of VLP vaccines depends on the adjuvant, purification and amount of the antigen [21,22,23]. In particular, antibody titers tend to correlate with the inoculated antigen dosage [23]. Neutralizing antibodies at high titers have important functions in the defense of the body against viral spreading by inhibiting attachment and infection of new cells [24]. As far as PCV2 vaccines are concerned, neutralizing antibodies can be the only factor to prevent clinical symptoms of PCV2. The absence of PCV2-neutralizing antibodies was correlated with high PCV2 replication and PCV2-associated disease [25]. Therefore, VLP dosage may be the main determinant of PCV2 vaccine efficacy. However, there is no report about the minimal dosage of VLP that is sufficiently potent to induce neutralizing antibodies as PCV2 vaccine antigen. Here, we suggested a novel basal line of VLP dosage based on two animal experiments using guinea pigs and conventional pigs. The guinea pig experiment was performed to confirm the presence or absence of antibodies produced by the VLPs themselves rather than quantitative estimation of induced antibodies, whereas the pig experiment indicated the practical minimal VLP dosage to protect conventional pigs against experimental challenge of PCV2d field isolate, which will be a useful reference for future vaccine development. Additionally, comparison of antibodies in guinea pig and pig models may provide a better understanding of pig immunity.

## 2. Material and Methods

### 2.1. PCV2d Capsid Expression and VLP Confirmation

Capsid protein of PCV2d was expressed from baculovirus infected Sf9 insect cells, as previously described [26]. Briefly, the PCV2d ORF2 gene (GenBank no. KM924369) was cloned into the pFastBac expression vector (Invitrogen, Carlsbad, CA, USA) and transfected into Sf9 cells. The culture supernatant containing the recombinant baculovirus was harvested and used to infect a separate batch of Sf9 cells. After 3 days, VLPs were purified from culture supernatant by sucrose gradient ultracentrifugation as previously described [26].

The VLP structure was confirmed by using a specific antibody to detect the decoy epitope (169–180 aa) on the inside of the particle, which may induce non-neutralizing antibodies wasting immunologic capacity [26]. For the process, the wells of a 96-well microtiter plate were coated with BSA-conjugated synthetic the decoy epitope 169–180 peptides, N terminal deleted capsid protein, or the purified VLP in coating buffer (0.1 M NaHCO_3_, pH 8.6) and then blocked with 3% (*w*/*v*) BSA in phosphate-buffered saline (PBS). After incubation with 1 mg/mL of the anti-CP169-180 antibody, horseradish peroxidase (HRP)- conjugated goat anti-human Ck antibodies (Chemicon-Millipore, Billerica, MA, USA) were added to each well. After washing with 0.05% (*v*/*v*) Tween 20 in PBS (PBST), 3,30,5,50-tetramethyl benzidine (TMB) (GenDEPOT, Barker, TX, USA) substrate solution was added and the absorbance was measured at 650 nm with a Multiskan Ascent microplate reader (LabSystems, Helsinki, Finland) [26].

### 2.2. The Vaccine Formulation with Carbopol Adjuvant

Carbopol^®^ 974P Polymer (Lubrizol, Cleveland, OH, USA) 400 mg was dissolved in 180 mL distilled water, then sterilized by autoclaving at 121 °C for 20 min and stored at 4 °C until further use. Before use, the Carbopol solutions were neutralized to pH 7.3 with 5N NaOH. The polymer and 20 mL of the purified capsid protein were mixed on a magnetic plate stirred at 150 rpm for 1 h. Finally, a 1 mL dose containing VLP of PCV2d (2 μg, 10 μg, 20 μg, or 60 μg) and 2 mg of Carbopol adjuvant was prepared and stored at 4 °C before use.

### 2.3. Animal Studies

Two separated animal experiments were designed for the study (Table 1).

(1) Pig challenge experiment: 4 weeks old, colostrum-fed, cross-bred, conventional piglets were purchased from a PCV2 seronegative farm. A total 30 piglets were tested for anti PCV2 antibodies and the only seronegative individuals were selected. They were confirmed as non-viremic for PCV2 by real-time polymerase chain reaction (PCR). The blood samples collected from the piglets were also examined for PRRSV infection using real-time PCR (Forward primer: 5’-GAAGAGAAACCCGGAGAAGC-3’; Reverse primer: 5’-GAAGAGAAACCCGGAGAAGC-3’) and PRRSV-specific antibodies were detected using ELISA (HerdCheck PRRS 3XR™, IDEXX Laboratories Inc., Westbrook, Maine, USA). All piglets were confirmed as PRRSV-free.

For the experiment, group were designed to be randomized, blinded and controlled. Twenty-four pigs were randomly divided into six groups (4 pigs per group). The Carbopol adjuvanted vaccines in combination with VLP 2 μg (group 1), VLP 10 μg (group 2), VLP 20 μg (group 3) and VLP 60 μg (group 4) was inoculated intramuscularly as 1.0 mL dose at 5 weeks of age in the right side of the neck. After 3 weeks of vaccination, the vaccinated pigs (groups 1, 2, 3 and 4) and non-vaccinated challenged pigs (group 5) were inoculated intranasally with 3 mL of a virulent PCV2d strain (SNUVR140004, Genebank No. KJ437506.1) at 1.0 × 10^4^ tissue culture infective dose of 50% [TCID_50_/mL]. The challenging virus was isolated from lymph node of a pigs which had been vaccinated with a commercial PCV2a vaccine and showed PCVAD in Korea. The negative control group (group 6) was remained unvaccinated and unchallenged throughout the experiment.

The pigs in each group were managed separately within the facility. Clinical examination and weighing were performed daily. Blood samples and nasal swabs were collected weekly. All pigs were euthanized for necropsy 35 days after vaccination (at 14 days of challenge) and superficial inguinal lymph nodes were collected for histopathology and immunohistochemistry.

(2) Guinea pig inoculation experiment: A total 20 Specific-pathogen-free 250 to 300 g outbred male Hartley strain guinea pigs were purchased from Charles River Breeding Laboratories. They were inoculated intramuscularly in the hind legs. The same formulated vaccines with the pig inoculation were administered as half dose (0.5 mL) with VLP 1 μg (group 1), VLP 5 μg (group 2), VLP 10 μg (group 3) and VLP 30 μg (group 4). Boosting vaccination was performed with the same administration 14 days after priming vaccination. Blood samples were collected 14 days after second vaccination.

All of the methods in these two experiments were approved by the BioPOA Institutional Animal Care and Use Committee.

### 2.4. Serum Antibody Test

Total antibodies against PCV2d from guinea pig serum were quantified with an indirect fluorescence assay (IFA) using the sera and PCV2-infected PK-15 cells, as previously described [27]. Anti-PCV2 IgG antibodies of pig serum were tested by a commercial enzyme-linked immunosorbent assay (ELISA) kit (SK105, BioChek, Reeuwijk, The Netherlands) according to the manufacturer’s instructions.

The serum virus neutralization (SVN) test was also performed as previously described [28] on 0, 21 and 35 days after vaccination. The challenge virus (SNUVR140004) was used for the assay. Neutralizing antibodies titers were expressed as the reciprocal of the highest serum dilution that completely blocked the infection in the PK15 cells compared with the virus control.

### 2.5. Clinical, Virological and Pathological Evaluation

The pigs were clinically monitored and scored based on their physical conditions ranging from 0 (normal) to 6 (severe) [29]. The body condition included respiratory (cough, dyspnea) and digestive (diarrhea) symptoms. Meanwhile, rectal temperatures were measured daily at the same time for 35 days after vaccination.

Challenged virus gene was detected from serum samples using the QIAamp DNA mini kit (Qiagen, Crawley, UK) and quantified as PCV2 genomic DNA copy numbers by real-time PCR [30].

The morphometric analysis of histopathological lesion was blindly evaluated as previously described [31]. Superficial inguinal lymph nodes were collected during necropsy for histopathological evaluation. Three blocks of tissues were collected from the lymph nodes of each pig and serially sectioned and prepared. Three slides were obtained from each block to allow scoring of the pathologic lesions using the NIH Image J 1.43 m program (http://rsb.info.nih.gov/ij (accessed on 9 November 2021)). For each slide, 10 fields were randomly selected and evaluated as follows: 0 (no lymphoid depletion or granulomatous replacement), 1 (mild lymphoid depletion), 2 (moderate lymphoid depletion), 3 (severe lymphoid depletion and granulomatous replacement). The PCV2 antigens were also detected by immunohistochemistry using a rabbit polyclonal anti-PCV2 antibody (1:200 in PBS containing 0.1% Tween 20) in the same tissue sections [32].

### 2.6. Statistical Analysis

Statistical analysis was conducted using GraphPad Prism (v5.0; GraphPad Software Inc., San Diego, CA, USA). Results are expressed as means and standard deviations of the indicated number of independent measurements. Statistical significance was determined using two-tailed unpaired Student’s *t* tests and non-parametric Mann–Whitney’s u test. One-way ANOVA followed by Tukey’s multiple comparison test was used to analyze the relationship among the groups. A value of *p* < 0.05 was considered significant.

## 3. Results

### 3.1. VLP Was Purified and Quantified

The capsid protein was clearly expressed from the insect cells which had been transfected with bacmid containing PCV2d cap gene. SDS-PAGE showed 23 kDa size single band and western blot confirmed it as capsid protein by using a monoclonal PCV2 capsid specific antibody [26]. The protein was quantified by the Bradford assay which is based on the reaction of coomassie brilliant blue with basic amino acid residues of the protein. Serially diluted VLP solutions were prepared and adjuvanted with the Carbopol.

VLP structure fitness was evaluated with the decoy epitope (169–180 aa) specific antibody. The ELISA result demonstrated absence of the exposed internal decoy epitope in the VLP solution, showing similar level with negative control and significantly lower level than the synthetic the decoy epitope peptides and N terminal deleted capsid protein coating wells. The result indicated structural fitness of the produced VLP.

### 3.2. The Total Antibodies Showed Significant Difference among the Inoculated Guinea Pig Groups

**Figure 1:**
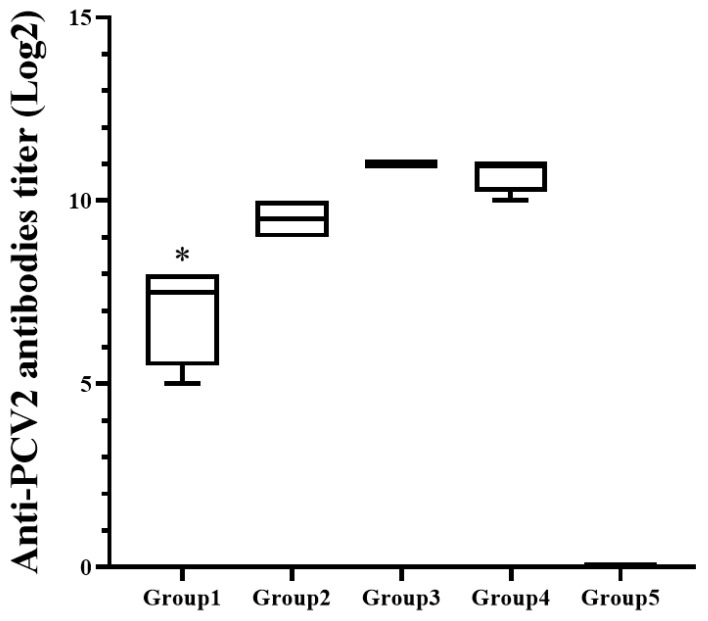
Mean titers of the anti PCV2 antibodies in different treatment guinea pigs. * indicates significant difference (*p* value < 0.05) between Group 1 vs. Group 2, 3, 4 and 5.

All blood samples of guinea pigs were collected 2 weeks after boosting. IFA result showed that the guinea pigs (Group 1) inoculated with 1 μg VLP had significantly (*p <* 0.05) lower titer of anti PCV antibodies than the other vaccinated guinea pigs (Group 2, 3 and 4) (Figure 1). However, it was significantly (*p* < 0.05) higher than titer of the negative control (Group 5). Guinea pigs (Group 2, 3 and 4) inoculated with greater than 5 μg VLP did not showed statistical difference for average antibody level. The negative control (Group 5) did not have any antibody throughout the experiment.

### 3.3. The Antibody Level of Vaccinated Pigs Was Significantly Higher Than That of Unvaccinated Controls

**Figure 2:**
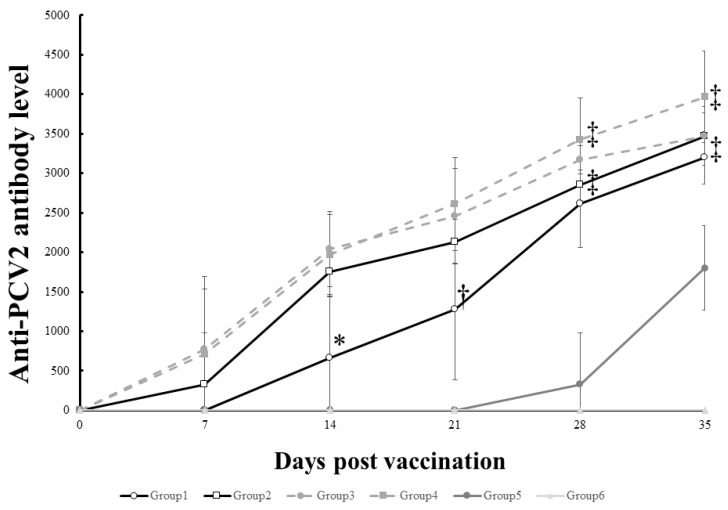
Mean levels of anti PCV2 IgG responses in different treatment pigs. * indicates significant difference (*p* value < 0.05) between Group 1 vs. Group 2, 3 and 4. † indicates significant difference between Group 1 vs. Group 3 and 4. ‡ indicates significant difference between Group 1, 2, 3 and 4 vs. Group 5 and 6.

Anti-PCV2 antibody results from the pig challenge experiment are summarized in (Figure 2). The vaccinated pigs started to seroconvert from 7 days after vaccination, whereas the non-vaccinated pigs (Group 5 and 6) remained seronegative until the challenge. The pigs (Group 2, 3 and 4) inoculated with greater than 10 μg VLP had significantly (*p <* 0.05) higher level of anti-PCV2 antibodies on 14 days after vaccination than the pigs (Group 1) inoculated with 2 μg VLP. At challenge, the pigs (Group 3 and 4) inoculated with greater than 20 μg VLP showed significantly (*p <* 0.05) higher antibody level compared to the pigs (Group 1) inoculated with 2 μg VLP. However, there was no significant difference in antibody level among the vaccinated groups on 7 days and 14 days after challenge. Unvaccinated control pigs (Group 5 and 6) showed significantly lower antibody level than the vaccinated pigs after the challenge.

### 3.4. The Neutralizing Antibodies Were Stably Detected in the Groups Inoculated with More Than 10 μg VLP

**Figure 3:**
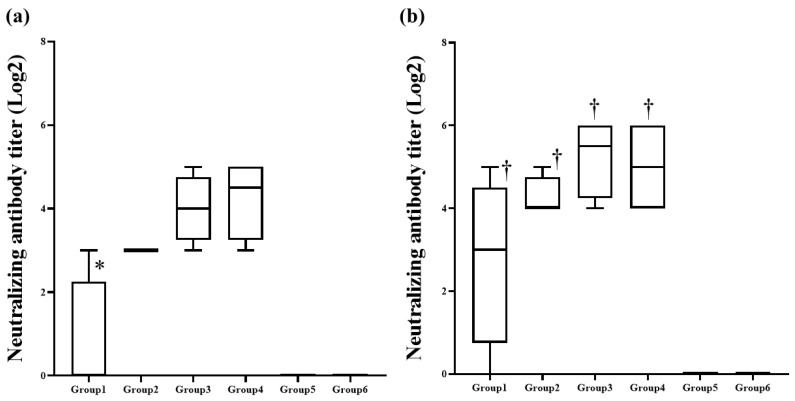
Mean titers of serum neutralizing antibodies in the different treatment pigs at 21 dpv, days post vaccination (**a**) and 35 dpv (**b**). * indicates significant difference (*p* value < 0.05) between Group 1 vs. Group 2, 3 and 4. † indicates significant difference between Group 1, 2, 3 and 4 vs. Group 5 and 6.

The neutralizing antibodies against the challenge PCV2d strain were sporadically detected in the vaccinated pigs. At 21 days after vaccination (the day of challenge), all pigs (Group 2, 3 and 4) inoculated with greater than 10 μg VLP had neutralizing antibody response to the challenging PCV2d strain, whereas the response was detected in the only one of four pigs (Group 1) inoculated with 2 μg VLP (Figure 3a). The neutralizing antibody titer of the Group 1 was significantly (*p <* 0.05) lower than that of the other vaccinated pigs (Group 2, 3 and 4). However, it was not statistically different with the unvaccinated controls (Group 5 and 6). 14 days after challenge, all vaccinated pigs (Group 1, 2, 3 and 4) had the neutralizing antibodies and mean titers of the groups were significantly (*p* < 0.05) higher than those of the unvaccinated controls (Group 5 and 6) (Figure 3b). Unvaccinated pigs did not show any neutralizing response throughout the experiment.

### 3.5. There Was No Difference in Average Daily Weight Gain (ADWG) between the Pigs Challenged with the Virulent PCV2d Isolate Regardless of Vaccination

Notable clinical signs and drastic increase of rectal temperature were not observed in any of the groups throughout the experiment. There was no significant difference in the mean clinical scores between vaccinated pigs (Group 1, 2, 3 and 4) and unvaccinated pigs (Group 5 and 6). The ADWG was also not significantly different between vaccinated and unvaccinated pigs throughout the experiment. All pigs had similar growth rate regardless of challenge (Table 2).

### 3.6. Viremia and Pathologic Lesion Score of Vaccinated Pigs Were Significantly Lower Than That of Non-Vaccinated Controls

**Figure 4:**
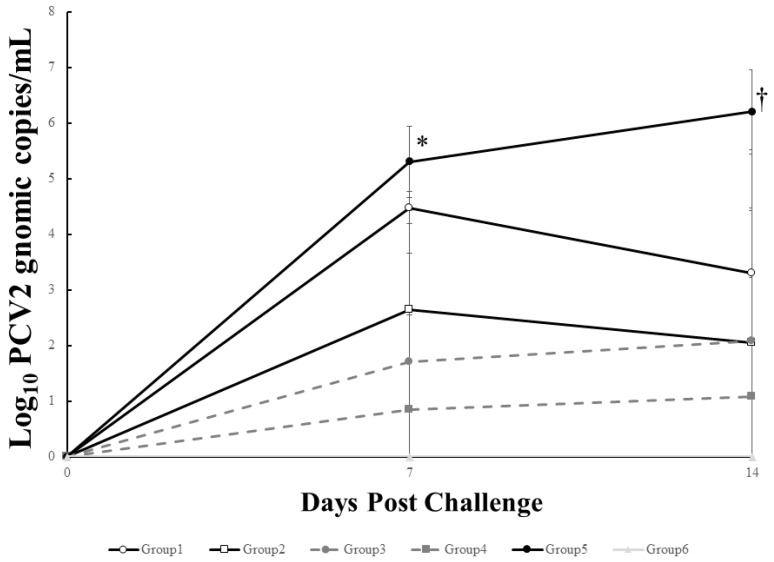
Mean genomic copy numbers of PCV2d DNA in serum from different groups. * indicates significant difference (*p* value < 0.05) between Group 5 vs. Group 2, 3, 4 and 6. † indicates significant difference between Group 5 vs. Group 1, 2, 3, 4 and 6.

At 7 days after challenge, the pigs (Group 2, 3 and 4) inoculated with greater than 10 μg VLP had significantly (*p <* 0.05) less genomic copies of the challenge virus than the unvaccinated challenged pigs (Group 5), whereas there was no significant difference on viral level between the pigs (Group 1) inoculated with 2 μg VLP and the control group (Group 5). 7 days after challenge, all vaccinated pigs (Group 1, 2, 3 and 4) had significantly (*p <* 0.05) less genomic copies than the unvaccinated challenged pigs (Group 5). There was also no significant difference on viral level between the vaccinated groups (Figure 4). PCV2 genomic DNA was not detected in any of unvaccinated unchallenged pigs (Group 6) throughout the experiment.

**Figure 5:**
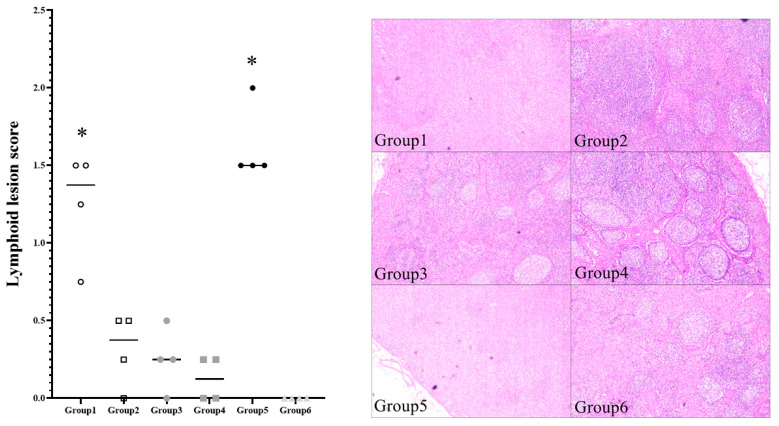
Histopathologic evaluation of lymphoid follicles from different groups on necropsy. * indicates significant difference (*p* value < 0.05) between Group 1 and 5 vs. Group 2, 3, 4 and 6.

The typical lymphoid depletion was observed in challenged pigs. Moderate to severe follicular depletion lesions were identified in not only unvaccinated challenged pigs (Group 5) but also the pigs (Group 1) inoculated with 2 μg VLP (Figure 5). Mild granulomatous inflammation reaction was also observed in individual challenged pigs and PCV2-antigen associated with the lesions was detected. The pigs (Group 2, 3 and 4) inoculated with greater than 10 μg VLP had significantly (*p <* 0.05) lower histopathologic lesion score than the unvaccinated challenged pigs (Group 5) and the pigs (Group 1) inoculated with 2 μg VLP. Group 2, 3 and 4 also had significantly less PCV2-positive cells per unit area in the lymph node compared to groups 1 and 5 (*p <* 0.05).

## 4. Discussion

PCV2 is one of the most economically important pathogens in the global swine industry. Various clinical signs associated with PCV2 infection have been reported, including wasting, increased mortality, respiratory signs, enteritis, dermatitis and reproductive disorders, in pigs of all ages [1,2,4,5]. The remarkable pathological feature of the infection is the enlargement of lymph nodes in affected pigs [6]. Severe lymphoid depletion, which is accompanied by diffuse infiltration of histiocytic cells, is also observed. Without vaccination, pigs exhibited aggravated weight loss, increased viremia level and severe lymphoid depletion lesions in a field study [33]. In the early 2000s, when the vaccine was not available, the mortality rate of affected pigs increased up to 80% [34].

Since commercial PCV2 vaccines have been launched, PCVAD has been receiving control in the field. The efficacy of commercial vaccines has been shown to be dependent on not only humoral immunity but also cell-mediated immunity, which can compensate for protection in the absence of detectable PCV2 antibodies in response to vaccination [35,36]. However, it is a neutralizing antibody that plays an important role in preventing virus entry into susceptible cells [25]. The elicited neutralizing antibody tends to correlate with the reduction of viral load from serum and tissue, indicating its protective effect against virus replication [25,27,35]. Therefore, an efficient vaccine is necessary to induce sufficient neutralizing antibodies to inhibit viral infection.

Most current commercial PCV2 vaccines are inactivated whole virus or subunit types, which contain the icosahedral virus particle as a major immunogen. The VLPs of subunit vaccines are reported to mimic the overall structure of the native virion and to be highly immunogenic, inducing humoral and cell-mediated immunity against PCVAD under field conditions [37]. The immunogen of subunit vaccines is commonly produced using the baculovirus expression system, which can secrete high levels of protein compared to porcine kidney-derived cell lines for whole virus production. The immunogen concentration in commercial vaccines can be dozens of times different depending on the vaccine type (data not shown).

In this study, we found that there was a baseline concentration in each animal model that elicited the antibody response, including neutralizing antibodies. The experiments suggested that 5 µg of VLP was essential to induce a stable humoral response in guinea pigs and 10 µg was needed in pigs. With injection into the intramuscular route, adjuvanted immunogens may be promptly exposed to blood and local immunity. Pathogen-associated molecular pattern motifs (PAMPs), which consist of the VLP, can trigger the activation of innate immune cells and be recognized by Toll-like receptors and other pattern-recognition receptors [38]. Subsequently, PAMPs can also stimulate the uptake of VLPs by antigen-presenting cells (APCs), especially dendritic cells. The antigen presentation of cells can lead to a strong adaptive humoral immune response [39,40,41]. Overall, the serial occurrence, including activation of the innate immune system and bridging with the adaptive response through presentation by APCs, may be based on the probability. A higher titer of antigens is more likely to be detected in multiple immune cells.

Antibodies against PCV2 were detected starting 1 weeks after vaccination. The titers of IgG and neutralizing antibodies were significantly higher in the pigs inoculated with more than 10 µg VLP than that in the other pigs 14 days after vaccination. After challenge, the neutralizing antibodies were still significantly higher in the pigs inoculated with more than 10 µg VLP, inhibiting viral replication in the serum and tissue. The pigs inoculated with more than 10 µg VLP showed significantly lower viremia and histopathological lesion scores than the unvaccinated challenged pigs. Meanwhile, clinical signs, weight gain and rectal temperature were normal in the groups regardless of vaccination, implying that the clinical symptoms caused by infection with the virulent PCV2d strain may be associated with several factors.

In conclusion, for the subunit vaccine type, 10 µg VLP is necessary to elicit stable neutralizing antibodies in pig models. It is considered to be a minimal dosage to trigger the immune system with the current formulation. The results may vary depending on the adjuvant, purification, antigenic fitness and inoculation route. All results can be useful and these data will be valuable for follow-up studies.

## Figures and Tables

**Table 1:** Group design of the animal experiments.

**Guinea Pig Experiment**						
	Group 1	Group 2	Group 3	Group 4	Group 5	
1st vaccination	1 μg VLP	5 μg VLP	10 μg VLP	30 μg VLP	None	
2nd vaccination	1 μg VLP	5 μg VLP	10 μg VLP	30 μg VLP	None	
**Pig Challenge Experiment**						
	Group 1	Group 2	Group 3	Group 4	Group 5	Group 6
Vaccination	2 μg VLP	10 μg VLP	20 μg VLP	60 μg VLP	None	None
Challenge	PCV2d	PCV2d	PCV2d	PCV2d	PCV2d	None

**Table 2:** Average daily weight gain (ADWG) from the different pig groups (group mean ± standard error).

	**Pig Challenge Experiment**					
	**Group 1**	**Group 2**	**Group 3**	**Group 4**	**Group 5**	**Group 6**
0~7 dpv						
ADWG	132 ± 21	442 ± 141	289 ± 374	157 ± 141	267 ± 133	310 ± 118
7~14 dpv						
ADWG	375 ± 86	414 ± 117	389 ± 102	428 ± 76	425 ± 114	464 ± 37
14~21 dpv						
ADWG	560 ± 101	410 ± 155	414 ± 192	621 ± 99	467 ± 44	582 ± 105
21~28 dpv						
ADWG	432 ± 147	653 ± 81	582 ± 119	553 ± 108	471 ± 144	564 ± 66
28~35 dpv						
ADWG	703 ± 131	514 ± 182	567 ± 97	635 ± 158	492 ± 245	621 ± 170

## Data Availability

The data present in the study are available on request from the corresponding author.
